# Effect of dietary fiber levels on bacterial composition with age in the cecum of meat rabbits

**DOI:** 10.1002/mbo3.708

**Published:** 2018-08-07

**Authors:** Zhenyu Wu, Hailiang Zhou, Fuchang Li, Nanbin Zhang, Yanli Zhu

**Affiliations:** ^1^ College of Animal Science and Technology Shandong Agricultural University Taian City Shandong Province China; ^2^ Shandong Provincial Key Laboratory of Animal Biotechnology and Disease Control and Prevention Shandong Agricultural University Taian City Shandong Province China; ^3^ College of Information Science and Engineering Shandong Agricultural University Taian City Shandong Province China; ^4^ Shandong Provincial Engineering Technology Research Center of Animal Disease Control and Prevention Shandong Agricultural University Taian City Shandong Province China

**Keywords:** cecal microbiota, high‐throughput sequencing, Neutral detergent fiber, rabbits

## Abstract

This study investigated the influence of dietary fiber levels on the growth performance, digestion, metabolism, and cecal microbial community of rabbits with different diets at different age. The different levels of dietary natural detergent fiber (NDF) were formulated accordingly: 400(A), 350(B), 300(C), 250(D) g/kg original matter basis, respectively; the different ages were 52, 62, and 72 days. With NDF increasing, the average daily feed intake (ADFI) and feed conversion rate (FCR) were increased, whereas average daily gain (ADG) and mortality were decreased (*p *<* *0.05). The stomach relative weight, stomach content relative weight, cecal relative weight, and cecal content weight increased with increasing NDF (*p *<* *0.05). The NH_3_‐N concentration of cecum dropped when the dietary NDF increased (*p *<* *0.05). The diversity of the total microbiota increased significantly in Diets B, C (*p *=* *0.011), and reached the lowest in 52 days for all diet groups. The richness index was decreased significantly in Diet A, D (*p *<* *0.05) and in 62 days (*p *<* *0.001), respectively. The phylum Firmicutes was higher (*p *<* *0.01) in rabbits fed Diets B, C than Diets A, D and Bacteroidetes was highest in Diets C, D, and Proteobacteria was the highest in Diet A (*p *<* *0.001). Among the classified genera, there were 14 that had levels of abundance of more than 1% and were commonly shared by all samples. *Ruminococcus* spp. that produced volatile fatty acid (VFA) abundance was highest from Diets B, C at 52 and 62 days. It is interesting to note that *Bifidobacterium* from Diet C was the most abundant genus during the entire experimental period (*p *<* *0.01). The data from Venn diagrams, principal component analysis (PCA), and heat map plots of the bacterial communities showed that there were more groups of shared microbiota with aging. The above results indicate the cecal microbiota controlled by the 350 g/kg NDF diet can prevent gastrointestinal distress and exhibit good production performance.

## 1 INTRODUCTION

The hindgut of monogastric animals harbors many thousands of microbial species (Daly, Stewart, Flint, & Shirazi‐Beechey, 2001; Ivarsson, Roos, Liu, & Lindberg, 2014). The cecum occupies 40% of the whole‐tract content size, and ecosystem of rabbits have highly active microbiota, which lead to a important role in their digestive physiology (Monteils, Cauquil, Combes, Godon, & Gidenne, 2008). The dietary fiber hydrolyzed by cecal microbiota results in the release of soluble sugars, which are fermented to VFA, such as acetate, propionate, and butyrate, etc. The VFA absorbed across the epithelium of the large intestine provide 30–50% of the rabbit's body energy (Gidenne, Pinheiro, & e Cunha, 2000). A previous study suggested that sufficient dietary fiber supply can prevent intestinal diarrhea in the growing rabbit (Gidenne, Arveux, & Madec, 2001). The recommended dietary NDF levels of rabbits are about 20%–34% (Gidenne, Jehl, Segura, & Michalet‐Doreau, 2002). Because of the main energy substrate for the microbiota, dietary fiber levels significantly impact of the composition of the intestinal microbiota (Combes, Fortun‐Lamothe, Cauquil, & Gidenne, 2013; Sawicki et al., 2015), and most of their beneficial effects are associated with bacterial fermentation (Lattimer & Haub, 2010; Verbeke et al., 2015). The understanding of cecal microbiota structure is considered to have an important role in the nutrition and health of rabbits.

The objectives of the research were to investigate and discuss the effects of different dietary NDF levels on the cecal ecosystem of growing meat rabbits and to determine the appropriate dietary NDF level in growing meat rabbits.

## 2 EXPERIMENTAL PROCEDURES

### 2.1 Animals, housing, and sampling

Two hundred weanling New Zealand rabbits were assigned into four groups according to the mean body weight (1.46 ± 0.16 kg). They were fed each diet with 400 (Diet A), 350 (Diet B), 300 (Diet C), and 250 (Diet D) g/kg NDF (original matter basis), respectively (Table 1). Rabbits were provided with free access to feed and a water nipple.

**Table 1:** Composition and nutrient levels of experimental diets (as fed basis) %

Item	Experimental diets			
	A	B	C	D
Corn	5.0	15.0	25.0	30.0
Wheat bran	5.0	10.0	15.0	20.0
Soybean meal	15.0	15.0	15.0	15.0
Corn germ meal	5.0	5.0	5.0	5.0
Medicago sativa	16.5	16.5	16.5	16.5
Soybean straw	12.0	6.0	0.0	0
Peanut vine	39.0	30.0	21.0	11.0
Bentonite	1.0	1.0	1.0	1.0
Salt	0.5	0.5	0.5	0.5
Vitamin‐mineral premix^a^	1.0	1.0	1.0	1.0
Total	100.0	100.0	100.0	100.0
Nutrient levels^b^				
GE (MJ/kg)	15.37	15.71	15.72	15.81
Dry matter	86.72	86.29	85.49	84.39
Crude protein	16.47	16.83	16.86	16.94
Ether extract (EE)	2.73	2.56	2.44	2.32
Crude fiber	22.37	18.46	14.32	10.03
Neutral detergent fiber (NDF)	41.84	37.36	31.72	25.24
Acid detergent fiber (ADF)	25.39	23.08	20.70	17.51
Acid detergent lignin (ADL)	7.88	6.90	6.10	5.19
Ash	10.28	9.35	8.20	7.30
Ca	0.94	0.95	0.84	0.81
P	0.75	0.80	0.71	0.73

Notes

a Premix provided the following per kg of diets: VA 8000 IU; VD 31,000 IU; VE 50 mg; Lys 1.5 g; Met 1.5 g; Cu 50 mg; Fe 100 mg; Mn 30 mg; Mg 150 mg; I 0.1 mg; Se 0.1 mg.

b Nutrient levels were measured values.

### 2.2 Production trial

All rabbits were weighed at the beginning (day 32) and the end (day 72) of the experimental period and the average daily gain (ADG), the average daily feed intake (ADFI), feed conversion rate (FCR), and mortality were recorded and calculated for the whole experimental period. The stomach weight, stomach content, cecal weight, cecal content weight, small intestine length, and rabbit body length were measured at 72 days, which were used for computing relative stomach weight (stomach weight/body weight), stomach content relative weight (stomach content weight/body weight), small intestine relative length (small intestine length/body length), cecal relative weight (cecal weight/body weight), cecal content relative weight (cecal content weight/body weight), mortality percentage (mortality number/rabbits total numbers per group). After slaughter, the cecum were removed immediately, cecal content (*n* = 8) used for measuring the pH value, NH_3_‐N concentration, VFA by the technique of Weatherburn (1967) and Tao and Li (2006).

### 2.3 Intestinal microbiota trial

Five healthy rabbits randomly selected from each diet treatment group were euthanized at 52, 62, and 72 days of age and harvested their cecal contents from the middle of the ccum, and stored at −80°C until microbial analyses.

Animal management and experimental procedures abided by the welfare guidelines of the Animal Care Committee, Shandong Agricultural University, People's Republic of China.

### 2.4 Chemical analysis of experimental diets

The diets were formulated according to the values of the National Research Council (NRC; 1977) and recommendations in “The Nutrition of the Rabbit” (De Blas et al.*,* 1998), and the food was formed the as pellets of 4 mm diameter (Table 1), and food or drinking did not add antibiotics in the total experiment. The experimental diets were analyzed dietary ingredient according to the recommendations of the Association of Official Analytical Chemists (National Standards Recommend Method, China). The crude fiber was measured by the acid‐base method, and the levels of NDF and acid detergent fiber (ADF) were determined using the detergent method developed by Van Soest (1963).

### 2.5 DNA extraction and purification

For each sample, DNA was obtained using a QIAamp^®^ DNA Stool Mini Kit (Qiagen, Hilden, Germany) according to the manufacturer's instructions. In order to avoid bias, DNA was extracted in duplicate (Michelland et al., 2010) for 16S rRNA sequencing. A_260/280_ measurement was used to assess the DNA quality by a DU640 Nucleic Acids and Protein Analyzer (Beckman Coulter, Brea, CA). DNA samples were sent to Hanyu Bio‐Tech (Shanghai, China) for V3–V4 region of the 16S rRNA gene high‐throughput sequencing with an Illumina MiSeq platform according to protocols described by previous studies (Caporaso et al., 2010).

### 2.6 Statistical and bioinformatic analyses

The data of production trial were subjected to one‐way analysis of variance (ANOVA) using the general linear models (GLM) procedure in SAS 9.1 (SAS institute Inc., Cary, NC). Intestinal microbiota richness and diversity indexes (Tables 5,6) were analyzed by one‐way or two‐way analysis of variance to examine the effects of diet, age, and diet×age. *p *<* *0.05 and *p *<* *0.01 indicates significant difference and highly significant difference among 16S sequences from different treatments, respectively. The valid sequences were filtered from the high‐throughput sequencing with standards previously reported (Hamady et al.*,* 2008). Briefly, an average quality score<25 and min length <200 bp were considered as low‐quality sequences, and any mismatches to the primers, or a homopolymer longer than 8 bases were excluded. The passing sequences were assigned to the individual sample according to the 7‐bp barcodes, and the remaining sequences were clustered into operational taxonomic units (OTUs) with a cutoff of 97% identity. Rarefaction curves, abundance‐based coverage estimates (ACE), Chao1 (Chao, Chazdon, Colwell, & Shen, 2005), network analysis, heat map analysis, and beta diversity analysis indexes were generated at 97% sequence identity level using RDP classifier. In addition, a principal component analysis (PCA) was performed based on weighted UniFrac distance. The diversity index (Simpson/Shannon), Good's coverage, and classification for all sequences were determined with Mothur (Schloss et al., 2009).

## 3 RESULTS

### 3.1 Effects of different dietary NDF on growth performance and total tract apparent digestibility and nitrogen balance

ADFI, ADG, FCR, and mortality during the growth trial are summarized in Table 2. ADFI and FCR were increased when NDF increasing (*p *<* *0.05). ADG trend is contrast with above. Mortality reached minimum levels in diet A and diet B (*p *<* *0.01). The stomach relative weight, stomach content relative weight, cecal relative weight, and cecal content weight increased with increasing NDF (*p *<* *0.05). The level of NDF did not affect small intestine relative length (Table 3).

**Table 2:** Effects of NDF level on growth performance of growing meat rabbits (*n* = 50)

Items	Diets				*p*‐value
	A	B	C	D	
Average daily feed intake (ADFI, g/d)	156.91 ± 4.88^a^	158.66 ± 4.64[Link]	142.62 ± 3.39^a^	138.45 ± 3.24[Link]	0.022
Average daily gain (ADG, g/d)	18.53 ± 2.90^B^	28.40 ± 2.33^A^	32.20 ± 2.20^A^	40.76 ± 2.29^C^	0.001
Feed conversion rate (FCR)	8.47 ± 0.26^A^	5.59 ± 0.16^B^	4.43 ± 0.11^C^	3.97 ± 0.21^C^	<0.001
Mortality (%)	1.7 ± 0.02^A^	1.5 ± 0.05^B^	4.8 ± 0.11^C^	11.2 ± 0.31^C^	<0.00 1

a Different small letter superscripts mean significant difference (*p *<* *0.05).

_A,B,C_ Different capital letter superscripts mean significant difference (*p *<* *0.01); whereas with the same or no letter superscripts mean no significant difference (*p *>* *0.05).

**Table 3:** Effects of NDF level on gastrointestinal development of growing meat rabbits (*n* = 8)

Items	Diets				*p*‐value
	A	B	C	D	
Stomach relative weight/%	6.85 ± 0.40^a^	6.17 ± 0.37^a^	5.30 ± 0.25^a^	5.12 ± 0.15^a^	0.012
Stomach content relative weight/%	4.87 ± 0.37^a^	4.38 ± 0.33a,b	3.64 ± 0.24^a^	3.22 ± 0.11^a^	0.031
Small intestine relative length	3.89 ± 0.28	3.81 ± 0.18	3.61 ± 0.19	3.38 ± 0.23	0.853
Cecal relative weight/%	8.84 ± 0.70^a^	7.76 ± 0.33a,b	6.93 ± 0.39^a^	6.56 ± 0.6^a^	0.022
Cecal content relative weight/%	4.87 ± 0.37^a^	4.38 ± 0.33a,b	3.64 ± 0.24^a^	3.49 ± 0.24^a^	0.031

Stomach relative weight = Stomach weight/body weight; Stomach content relative weight = Stomach content weight/body weight; Small intestine relative length = Small intestine length/body length; Cecal relative weight = Cecal weight/body weight; Cecal content relative weight = Cecal content weight/body weight.

_a,b_ Different small letter superscripts mean significant difference (*p *<* *0.05), whereas with the same or no letter superscripts mean no significant difference (*p *>* *0.05).

The effect of different dietary NDF on cecal fermentation is shown in Table 4. The only NH_3_‐N concentration of cecum dropped when the dietary NDF increased (*p *<* *0.05).

**Table 4:** The cecal fermentation character of experimental rabbits (*n* = 8)

Items	Diets				*p*‐value
	A	B	C	D	
Total volatile fatty acid (VFA, mmol/L)	38.23 ± 3.13	41.97 ± 2.73	43.76 ± 3.69	44.07 ± 2.69	0.325
Propionic acid proportion (100%)	4.14 ± 0.63	3.85 ± 1.02	5.43 ± 2.02	5.32 ± 0.53	0.395
Acetic acid proportion (100%)	80.43 ± 0.88	78.98 ± 0.78	76.16 ± 0.58	74.76 ± 0.59	0.069
Butyric acid proportion (100%)	15.43 ± 1.68	16.18 ± 0.98	18.41 ± 1.68	20.12 ± 0.73	0.051
NH3‐N (mmol/L)	21.22 ± 1.13^a^	22.36 ± 0.55a,b	23.56 ± 0.48a,b	26.18 ± 0.57a,b	0.033
pH value	6.82 ± 0.03	6.73 ± 0.04	6.56 ± 0.07	6.53 ± 0.05	0.074

Notes

_a,b_Different small letter superscripts mean significant difference (*p *<* *0.05), whereas with the same or no letter superscripts mean no significant difference (*p *>* *0.05).

### 3.2 Illumina MiSeq derived metadata

**Figure 1:**
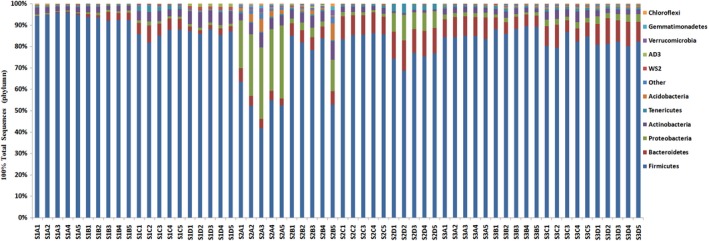
Bacterial composition of the different diet treatments at the phylum level. A, B, C, D represented the sequences of cecum from Diet A, B, C, D, respectively. A1, A2, A3, A4, A5……D5 represented five repeat per diet treatment, respectively; S represented sampling time at 52d (S1), 62d (S2), 72d (S3). The same as below

The raw sequences with an average length of 402 bp were obtained from the Illumina Miseq platform. By quality filtering, the obtained valid sequences per sample (based on 97% sequence identity) were aligned in accordance with the RDP classifier alignment and clustered into OTUs. These OTUs were assigned to 10 different phyla (Figure 1).The rarefaction curves of all samples are shown in Supplementary Figure S1. Although the corresponding curve from each sample was not plateaued indicating that complete sampling in rabbits cecum had not yet been achieved, Good's coverage estimates pointed out a large part of the diversity in all samples captured with an average coverage of 96.2% (data not shown).

### 3.3 Microbial diversity and abundance

The Shannon–Weaver diversity index was calculated to assess the diversity of the microbial communities. Using two‐factor analysis, the DNA sequences from all five rabbits in each diet group were used individually for these calculations, and diversity indexes were compared over time among different diets (Table 5). The diversity of the total microbiota decreased significantly in diets A and D (*p *=* *0.011), and the diversity levels reached the lowest in 52 days for all diet groups (Table 2, *p *<* *0.001). The numbers of OTUs, the Chao 1, and ACE estimates at two cut‐off levels of 3% are provided in Supplementary Table S1, which showed OTUs number, Chao 1, and ACE, associated with richness significantly increased in Diets A, B (*p *=* *0.03, *p *=* *0.003, *p *=* *0.002, respectively); the richness indexes for these diet groups significantly increased in 62 days (Table 2, *p *<* *0.001).

**Table 5:** Indexes^a^ of microbial richness and diversity for all samples

	Diet				Age (days)				*p* level		
	A	B	C	D	52	62	72	RMSE	Diet	Age	Diet × Age
Shannon index	5.44a,b	5.67^a^	5.56^a^	5.28^b^	5.13^B^	5.71^A^	5.63^A^	0.33	0.011	<0.001	<0.001
ACE	15300[Link]	17890^A^	2970^B^	3290^B^	2016^B^	22729^A^	4843^B^	12409.43	0.002	<0.001	<0.001
Chao 1	8561^A^	10044^A^	2651^B^	2672^B^	1817^B^	12361^A^	3769^B^	6552.36	0.003	<0.001	<0.001
OTUs	3134.8^ba^	3573.9^a^	1665.1^b^	1564.3^b^	1250.1^B^	4196.3^A^	2007.3^B^	2200.33	0.030	<0.001	0.005

RMSE, Root mean square error; ACE, abundance‐based coverage estimates.

a The operational taxonomic units (OTUs) were defined at 3% dissimilarity level. The richness estimators (ACE and Chao1) and diversity indexes (Shannon) were calculated using the Mothur analysis.

_a,b_Different small letter superscripts mean significant difference (*p *<* *0.05);

_A,B,C_ Different capital letter superscripts mean significant difference (*p *<* *0.01); whereas with the same or no letter superscripts mean no significant difference (*p *>* *0.05).

### 3.4 Taxon‐based analysis and Phylum‐level difference in microbial composition among group

To describe the bacterial composition of the different dietary treatment groups and how they changed during total experimental period, we conducted a taxon‐dependent analysis (Cole et al., 2013). Describing the distribution of DNA sequences at phylum level in Figure 1, the results showed that bacterial communities of all samples were composed primarily of Firmicutes, Bacteroidetes, Proteobacteria, and Actinobacteria, which had overall majority of the total sequences. Significant fluctuations of Firmicutes, Bacterioidetes, Proteobacteria, and Actinobacteria were detected among the four diets and three ages (Table 6). The phylum Firmicutes was higher (*p *<* *0.01) in rabbits fed Diets B and C when compared with the other two diets (Diets A and D). The phylum Bacteroidetes was higher when rabbits were fed with Diets C and D than the other diets (*p *<* *0.001). The highest level of NDF (Diet A) increased the Firmicutes/Bacteroidetes ratio (*p *<* *0.001), and a higher Firmicutes/Bacteroidetes ratio occurred in the 52‐d age group (*p *<* *0.001). However, phylum Proteobacteria was the highest in Diet A (*p *<* *0.001). Similarly, means from each age group among the four diets were compared by an analysis of diet × age (Table 6). For all of the above bacterial phyla, the interaction between diet and age was highly significant (*p *<* *0.001).

**Table 6:** Effect of different diets on Relative abundance of the main bacterial phyla^a^

	Diet				Age (days)				*p* level		
	A	B	C	D	52	62	72	RMSE	Diet	Age	Diet × Age
Firmicutes	77.43^B^	85.77^A^	84.49^A^	80.80^B^	90.02^A^	72.22^C^	84.12^B^	4.8	<0.001	<0.001	<0.001
Bacteroidetes	4.70^B^	4.76^B^	7.59^A^	8.32^A^	2.87^B^	8.01^A^	8.13^A^	1.147	<0.001	<0.001	<0.001
Firmicutes/Bacteroidetes^a^	126.89^A^	25.62^B^	12.04^B^	18.09^B^	115.57^A^	10.1^B^	11.31^B^	80.35	<0.001	<0.001	<0.001
Proteobacteria	10.71^A^	2.63^C^	1.76^C^	4.44^B^	1.00^B^	11.36^A^	2.30^B^	2.12	<0.001	<0.001	<0.001
Actinobacteria	3.37	3.44	3.50	3.17	3.78^A^	3.50^A^	2.82^B^	0.75	_	<0.001	<0.001

a Relative abundance of a phylum in different libraries was calculated as percentage of the sequence of this phylum to all sequences in that sample.

Firmicutes/Bacteroidetes represented the their percentage of the sequence ratio.

b Different capital letter superscripts mean significant difference (*p *<* *0.01); whereas with the same or no letter superscripts mean no significant difference (*p *>* *0.05).

### 3.5 Genus‐level differences in microbial composition among groups

**Figure 2:**
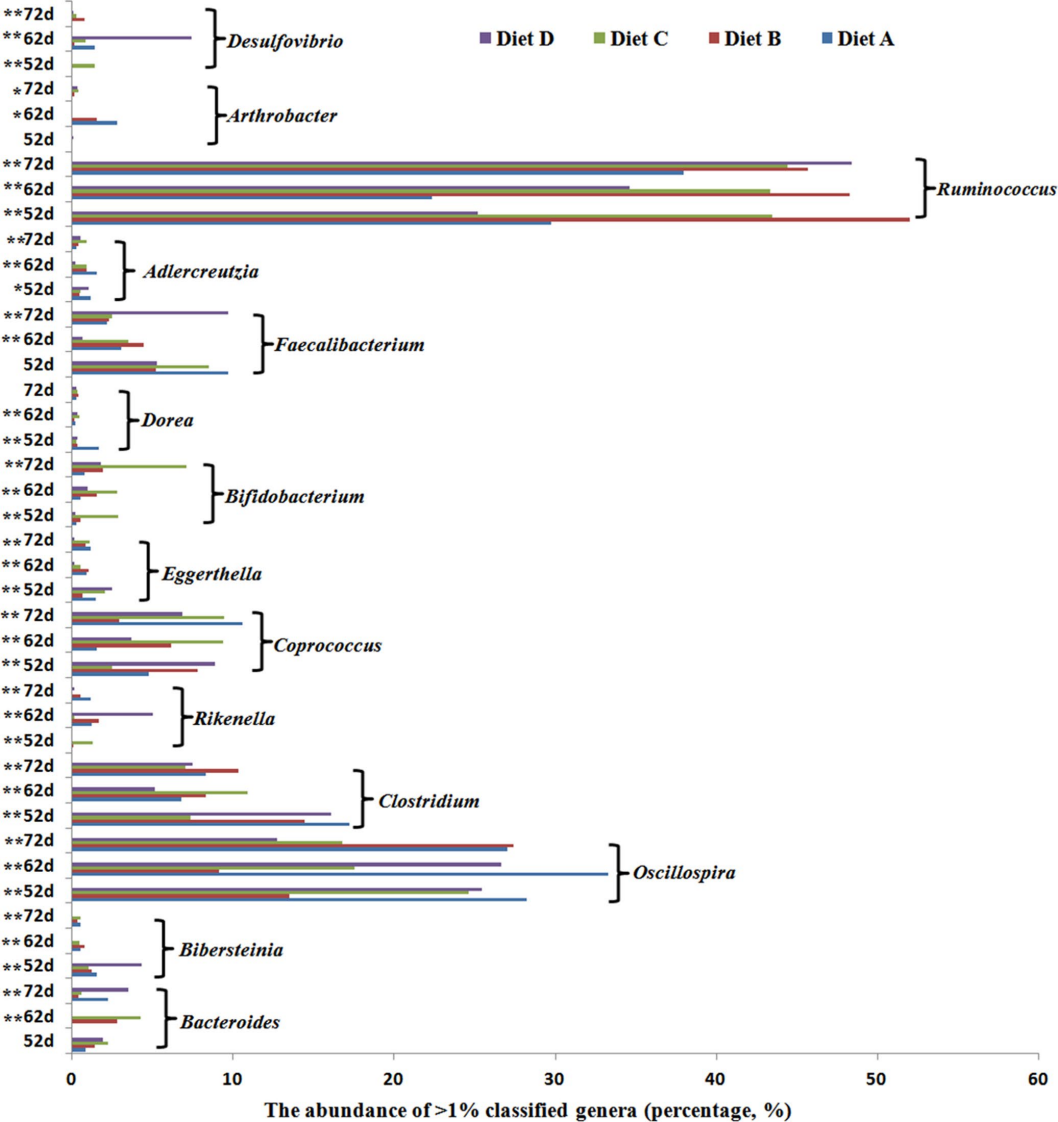
The histogram represented occurred at >1% abundance in at least one sample at genus level, and their comparing among four different NDF levels diets in 52, 62, and 72 days

As shown Figure 2, among the classified genera, there were 14 predominant genera (>1%) and were commonly shared by all samples (in any sample): *Desulfovibrio, Arthrobacter, Ruminococcus, Adlercreutzia, Faecalibacterium, Dorea, Bifidobacterium, Eggerthella, Coprococcus, Rikenella, Clostridium, Oscillospira, Bibersteinia,* and *Bacteroides*. Comparing the different diet treatments in rabbits of the same age shows that except for *Bacteroides* and *Faecalibacterium* at 52 days and *Dorea* at 72 days, the abundance levels of the above bacteria genera were significantly different among the four dietary treatments at 52, 62, and 72 days (*p *<* *0.05). *Ruminococcus* was the most abundant genus among the four treatments, accounting for 22%–52% of the total number of high‐quality bacterial sequences. Moreover, the levels of *Ruminococcus* spp. differed significantly among the four treatments at the same sampling time (Figure 2). Except for the 72‐day sample, the *Ruminococcus* spp. abundance was highest in Diets B and C at 52 and 62 days. It is interesting to note that *Bifidobacterium* from Diet C was the most abundant genus during the entire experimental period (*p *<* *0.01). However, the trends were similar to the abundance levels of *Desulfovibrio* in Diet C samples. *Clostridium* only occurred at very low levels (*p *<* *0.01) in Diet C, except for the 62‐day samples; it was most abundant in rabbits fed Diet A at 52 days (*p *<* *0.01) and Diet B at 72 days (*p *<* *0.01). *Oscillospira* accounted for 6%–41% of the total bacterial effective sequences in all samples, whereas *Oscillospira* was more abundant in Diet A than the other diet groups in the 52‐ and 62‐day samples (*p *<* *0.01) and was extremely highly abundant in rabbits fed Diets A and B in the 72‐day samples (*p *<* *0.01). The distribution of some abundant genera also changed with aging. For instance, the genus *Coprococcus* was more abundant in rabbits fed Diets B and D at 52 days (*p *<* *0.01), Diet C at 62 days (*p *<* *0.01) and Diets A and C at 72 days (*p *<* *0.01). *Bibersteinia* was highly abundant in rabbits fed Diet D (4.36%, *p *<* *0.01) at 52 days, but was no longer abundant (0.09%, *p *<* *0.01) at 62 and 72 days. The sequences assigned to *Faecalibacterium* were significantly affected by diets of different fiber levels (62 and 72 days, *p *<* *0.01), whereas Diets B and C elicited higher percentages of total bacterial effective sequences at 62 days (4.5%). In addition, Diets A and C elicited a high abundance of *Faecalibacterium* at 62 days (3.52%; 3.07%) and Diet D (9.72%) at 72 days.

### 3.6 Relationships of cecal bacterial communities feeding different dietary fiber levels

**Figure 3:**
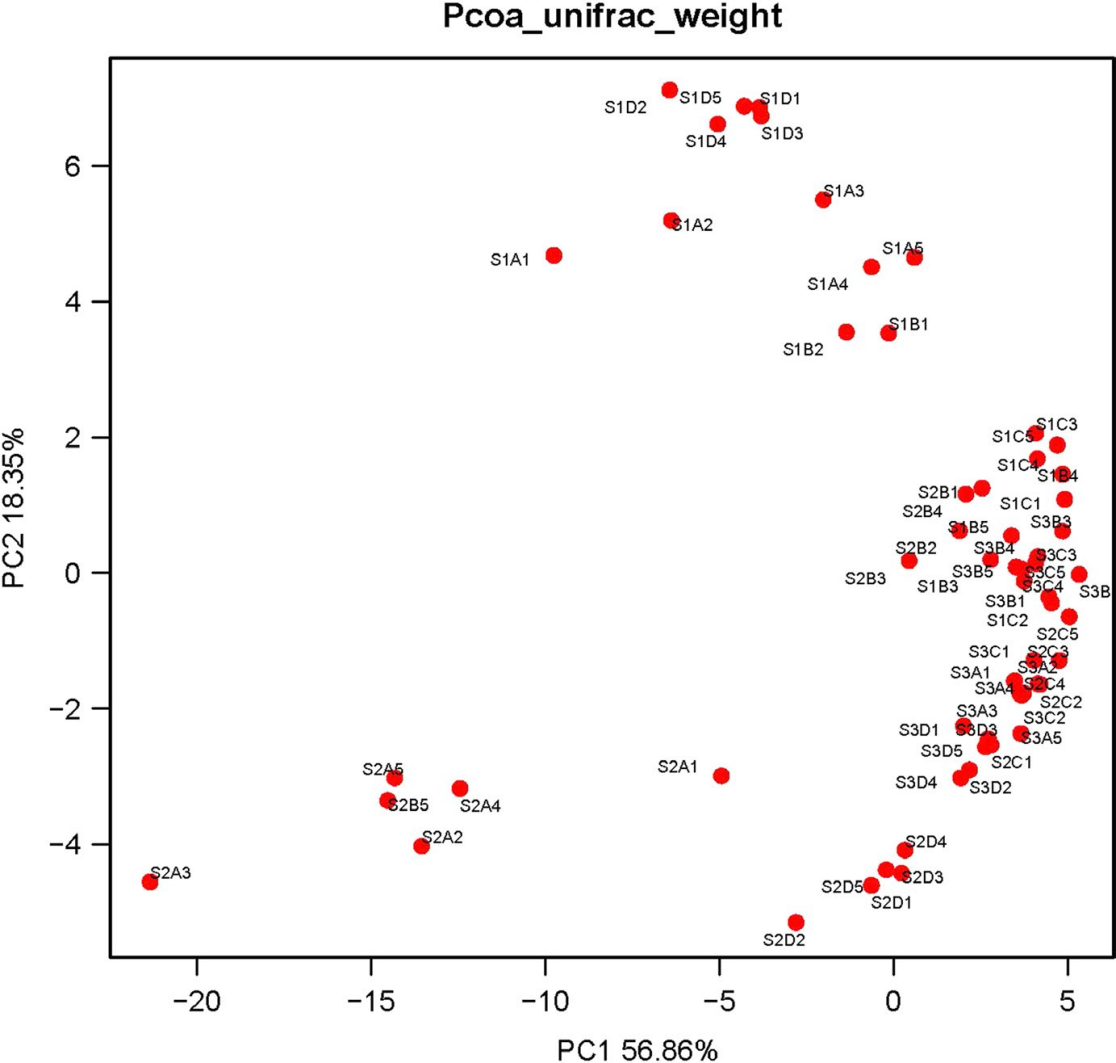
Sample sorting analysis

**Figure 4:**
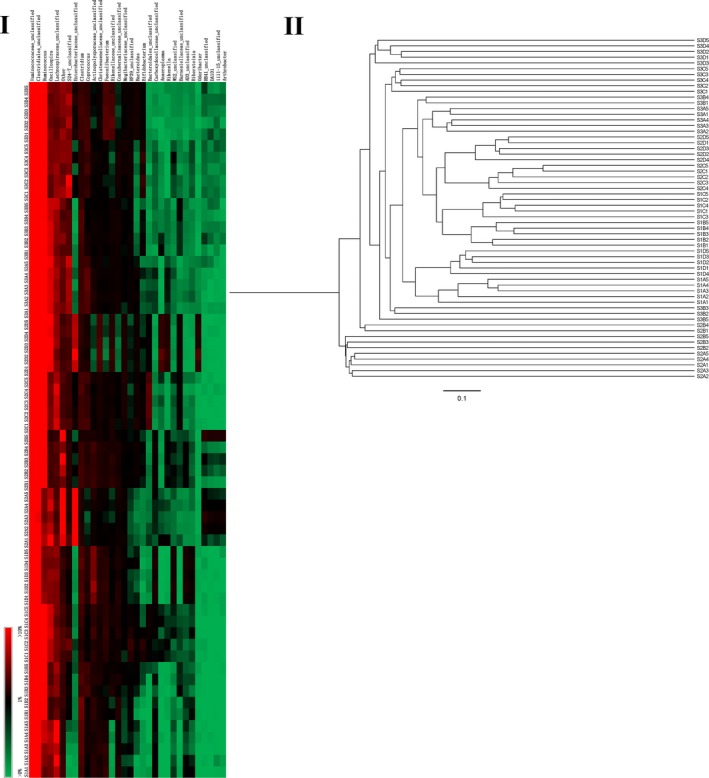
Bacterial distribution among all samples

The principal component analysis of the weighted UniFrac distance matrix and heat map analysis were conducted to further evaluate the pyrosequencing data. The PCA score plot revealed that the majority of the samples harbored characteristic bacterial communities, and the samples from Diet C at 52 days (S1C1, S1C2, S1C3, S1C4, S1C5), Diets C and D at 62 days (S2C1, S2C2, S2C3, S2C4, S2C5), and Diets A, B, C and D at 72 days (S3A1, S3A2, S3A4, S3A5, S3B1, S3B2, S3B3, S3B4, S3B5, S3C1, S3C2, S3C3, S3C4, S3C5, S3D1, S3D2, S3D3, S3D4, S3D5) grouped to the right of the graph along PC1, which accounts for 56.86% of the total variations. Others were separate from the above samples along PC2, which represented 18.35% of the total variations (Figure 3). Overall, the two PCA axes explained 75.21% of the variation between the different communities. Analysis of the hierarchically clustered heat map based on the bacterial community profiles at the genus level revealed the same trend (Figure 4).

**Figure 5:**
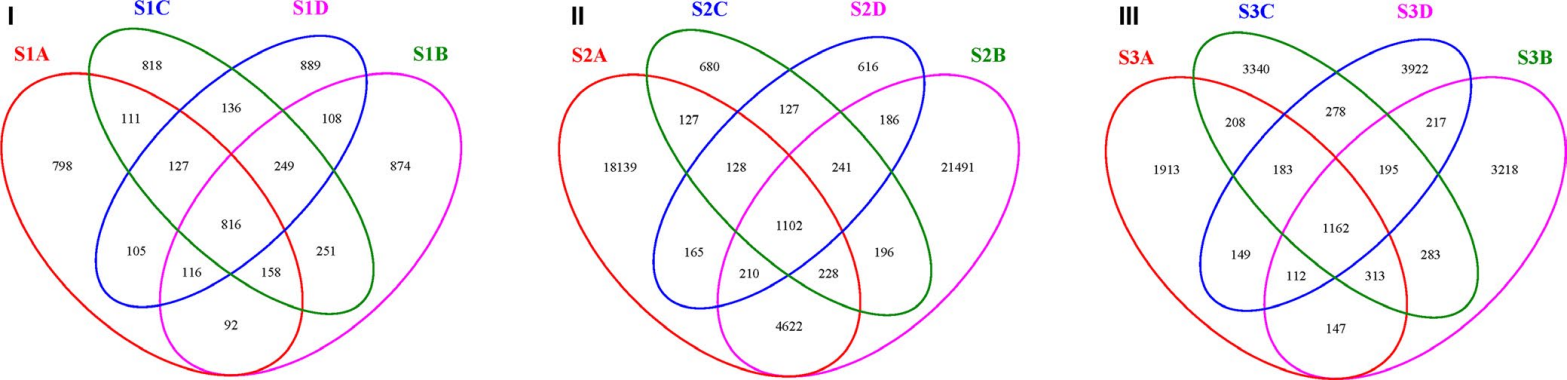
Shared OTUs analysis of the different libraries. Venn diagram showing the unique and shared OTUs (3% distance level) among the different libraries in 52 days (I), 62 days (II), and 72 days (III)

Each Venn diagram represents the sum of genus‐level OTUs at each sampling time point of each sample (Figure 5). Examination of these genera captured the shared and unique genus‐level OTUs of all samples in the same age. Comparative analysis indicated 816, 1102 and 1162 OTUs shared by different dietary treatments at 52, 62, and 72 days, respectively. Therefore, the number of shared OTUs among different treatments tended to increase with age.

## 4 DISCUSSION

Although the rabbit relies on the intake of large quantities of fiber that can be fermented by the microbiota found predominantly within the cecum, the change in NDF level does not affect the cecal pH and VFA concentration, which indicates no relation between quantity of NDF and concentration of VFA in the cecum (Bellier & Gidenne, 1996). Previous studies focused particularly on the analysis of the effects of the dietary fiber level on the whole cecal bacterial community in rabbits using molecular profiles (Michelland et al., 2010, 2011; Monteils et al., 2008; Rodríguez‐Romero, Abecia, & Fondevila, 2013). Our study showed that the cecal microbiota abundance increased significantly when NDF levels reached 350–400 g/kg (original matter basis) (*p *<* *0.001). However, 400 g/kg and 250 g/kg NDF groups decreased the diversity of the cecal microbiota (*p *=* *0.01). Therefore, feeding rabbits with different dietary fiber levels resulted in alteration of the structure of the cecal bacteria community (diversity and relative abundance) (Crowley et al., 2017). We also noticed that the cecal bacterial diversity increases with age, whereas bacterial abundance maintains a dynamic balance (in this case, increased first and then decreased).

We found that more than 90% of DNA sequences of the cecal contents from all samples in the total experiment period belong to the Firmicutes, Bacteroides, Actinobacteria, and Proteobacteria, which is consistent with the findings of Cauquil and Gidenne (2012). In this study, rabbits feeding on high‐fiber diets (Diet A) required high cecal ratio of Firmicutes/Bacteroidetes (*p *<* *0.001). Combes et al. (2011) reported ratio Firmicutes/Bacteroidetes was proposed as an indicator for microbiota maturity. Thus, our results indicated that the higher NDF level diet can accelerate the maturation of intestinal microbiota. In addition, the mice and human studies suggest that a higher Firmicutes/Bacteroidetes impels the gut microbiota to extract efficiently from the diet, which reveal one cause of adiposity (Marcobal et al., 2011; Ley, Lozupone, Hamady, Knight, & Gordon, 2008; Yasuda et al., 2015). However, it is clearly established that an increase in NDF level results in a reduction in the ADG in our study (*p *<* *0.001), which apparently contradicts previous results. Here the result seems to be more related with physiological conditions, such as the rabbit cecum is different significantly from mice and human.

*Ruminococcus*,* Coprococcus*,* Oscillospira, Bifidobacteria,* and *Clostridium* were known as VFA‐producing genera, interestingly, our study also suggests that Only *Coprococcus* and *Oscillospira* richness was positively correlated with NDF levels. In fact, the quantity of all kinds of VFA among different treatments had no difference. But the effect of NDF levels on ADFI significantly increased with NDF reducing (*p *<* *0.01). More research will be needed to understand the relation between production and various microbial groups in the cecum. *Bifidobacterium* species are frequently associated with health‐promoting effects in the human and animal intestinal tracts (Arboleya, Watkins, Stanton, & Ross, 2016; O'Callaghan & van Sinderen, 2016; Combes et al., 2013). Notably, the 300 g/kg NDF group (Diet C) led to significantly increased *Bifidobacterium*. Additionally, we observed what appears to be a substrate‐related stimulatory effect of Diet C on *Desulfovibrio spp*. (producers of toxic sulfides). Inness, McCartney, Khoo, Gross, and Gibson (2007) reported, also, modulation of by increasing *Bifidobacteria* and decreasing *Desulfovibrio spp*. may be beneficial to cats with IBD. Moreover, in our study, the diarrhea index was found to be significantly higher in Diets C and D than in Diets A and B (*p *<* *0.001, data not shown), which indicated a positive relation with higher *Desulfovibrio spp*. (Diet C and Diet D, respectively, in 52 and 62 days, *p *<* *0.001, Figure 2). In additional, our study showed that rabbit production performance was elevated, that is, the ADG reached the highest levels in Diets C and D (*p* = 0.001, Table 2). This result indicated that abundance of *Desulfuricans spp*. may reflect elevated production (Luo et al., 2018). On the other hand, *Bibersteinia* is an important pathogen that is associated with serious infection (Bleich, Sargeant, & Wiedmann, 2018; Parr, Smith, Jenks, & Thompson, 2018; Heinse, Hardesty, & Harris, 2016) and is implicated in the high incidence of diarrhea in Diet D at 52 days.

Venn diagrams, PCA, and heat map plots of the bacterial communities derived from rabbit cecum showed that a shift of dietary fiber can lead to quick changes in the composition of the microbiota, which is a reflection of a rapid adaptation to reach a new equilibrium in response to a nutritional disturbance (Michelland et al., 2011; Zhu, Sun, Wang, & Li, 2017). Meanwhile, we found that in the cecum, the structure of the bacterial community has no difference between rabbits of the same age or fed the same diet treatment (Michelland et al., 2010).

## 5 CONCLUSION

We provide clear evidence that the influence of dietary NDF levels on growth performance, digestibility and metabolism, the cecal bacterial community differed between rabbits of different ages. Indeed, better production performance, increasing the cecal microbiota diversity and abundance were all benefited from suitable dietary NDF level in Diet B (350 g/kg NDF, original matter basis).

## CONFLICT OF INTEREST

All authors declare that there are no conflicts of interest.

## Data Availability

MiSeq Illumina sequencing raw sequence reads data: https://www.ncbi.nlm.nih.gov/bioproject/PRJNA291670, which associated with the “Illumina MiSeq derived metadata” section in the article.
