# Supplementary figures and images for: Effect of dietary fiber levels on bacterial composition with age in the cecum of meat rabbits
Source: Microbiologyopen. 2018 Aug 7;8(5):e00708. doi: 10.1002/mbo3.708 (PMC6528572; doi:10.1002/mbo3.708)

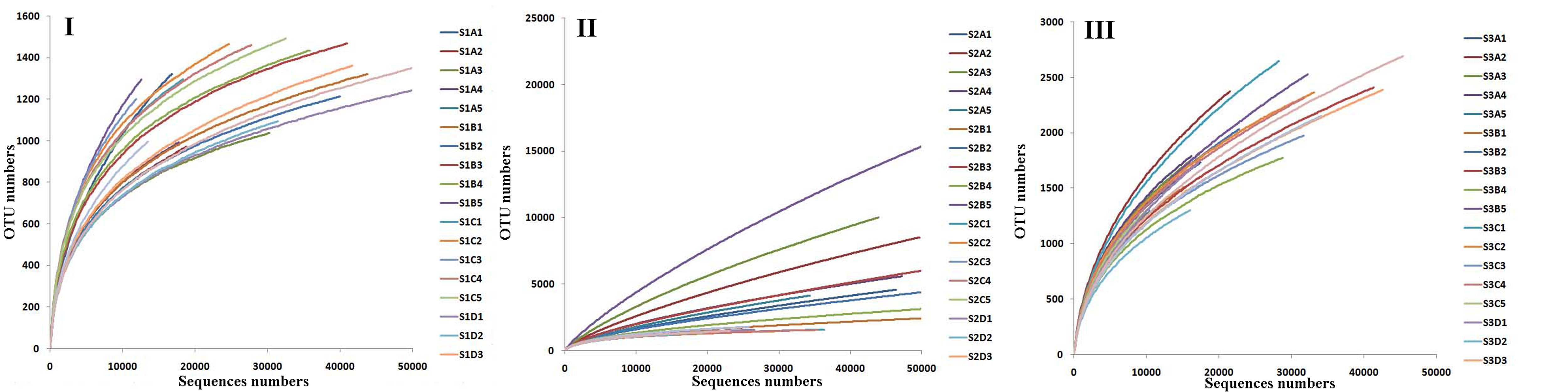

Supplement: Supplementary file 1 [file MBO3-8-e00708-s001.tif]
