# Supplementary material for: Effect of dietary fiber levels on bacterial composition with age in the cecum of meat rabbits
Source: Microbiologyopen. 2018 Aug 7;8(5):e00708. doi: 10.1002/mbo3.708 (PMC6528572; doi:10.1002/mbo3.708)
Supplement: Supplementary file 2 [file MBO3-8-e00708-s002.doc]

**TABLE S1.** Richness and diversity estimation for caecum bacterial populations based on alpha diversity analysis

| Sample  name^a^ | Valid sequences | OTU | ACE | Chao 1 | Shannon | Simpson |
| --- | --- | --- | --- | --- | --- | --- |
| S1A1 | 16777 | 1320 | 2862.09 | 2172.86 | 4.98 | 0.031612 |
| S1A2 | 18781 | 970 | 1648.00 | 1501.01 | 4.82 | 0.033929 |
| S1A3 | 30247 | 1036 | 1311.24 | 1315.101 | 4.80 | 0.035492 |
| S1A4 | 17726 | 991 | 1420.71 | 1454.64 | 4.82 | 0.037849 |
| S1A5 | 21181 | 996 | 1370.79 | 1440.024 | 4.88 | 0.030564 |
| S1B1 | 43742 | 1320 | 1778.97 | 1857.47 | 4.88 | 0.039274 |
| S1B2 | 40021 | 1212 | 1603.39 | 1684.96 | 4.75 | 0.041448 |
| S1B3 | 40983 | 1467 | 1866.99 | 1906.47 | 5.36 | 0.020719 |
| S1B4 | 35823 | 1434 | 1873.04 | 1992.69 | 5.46 | 0.018494 |
| S1B5 | 12562 | 1293 | 2597.91 | 2142.03 | 5.56 | 0.016248 |
| S1C1 | 18349 | 1295 | 2064.80 | 1902.07 | 5.55 | 0.01692 |
| S1C2 | 24649 | 1464 | 1921.03 | 1955.26 | 5.60 | 0.015585 |
| S1C3 | 11855 | 1199 | 2118.89 | 1856.27 | 5.56 | 0.016071 |
| S1C4 | 27749 | 1460 | 1899.35 | 1916.23 | 5.59 | 0.014849 |
| S1C5 | 32520 | 1491 | 1979.89 | 2098.63 | 5.58 | 0.015344 |
| S1D1 | 50893 | 1249 | 1989.62 | 1816.99 | 4.92 | 0.037095 |
| S1D2 | 31394 | 1093 | 1488.31 | 1500.96 | 4.84 | 0.036584 |
| S1D3 | 41728 | 1361 | 4327.97 | 2083.3 | 4.89 | 0.041665 |
| S1D4 | 13519 | 996 | 2012.75 | 1781.30 | 4.99 | 0.033868 |
| S1D5 | 50502 | 1354 | 2179.02 | 1952.73 | 4.84 | 0.039826 |
| S2A1 | 46402 | 4554 | 35783.81 | 16855.54 | 5.35 | 0.041139 |
| S2A2 | 49678 | 8496 | 56560.80 | 27345.04 | 5.90 | 0.044414 |
| S2A3 | 43916 | 9983 | 54122.75 | 30221.50 | 6.31 | 0.044962 |
| S2A4 | 47239 | 5570 | 27190.76 | 16791.39 | 5.50 | 0.050769 |
| S2A5 | 34353 | 4117 | 26973.10 | 13246.99 | 5.12 | 0.071687 |
| S2B1 | 58337 | 2603 | 8937.81 | 6789.76 | 5.60 | 0.014265 |
| S2B2 | 53996 | 4606 | 37491.27 | 18684.53 | 5.96 | 0.011681 |
| S2B3 | 53107 | 6258 | 64263.31 | 26788.29 | 6.19 | 0.010776 |
| S2B4 | 57870 | 3377 | 15454.87 | 9856.26 | 5.86 | 0.011452 |
| S2B5 | 70863 | 19794 | 107753.64 | 59885.25 | 7.84 | 0.004374 |
| S2C1 | 36366 | 1563 | 2063.34 | 2144.40 | 5.55 | 0.01692 |
| S2C2 | 30318 | 1444 | 1922.28 | 1988.47 | 5.48 | 0.015585 |
| S2C3 | 33220 | 1518 | 2322.52 | 2116.13 | 5.48 | 0.016071 |
| S2C4 | 35384 | 1532 | 2029.84 | 2105.83 | 5.48 | 0.014849 |
| S2C5 | 19967 | 1350 | 1852.83 | 2018.80 | 5.46 | 0.015344 |
| S2D1 | 26497 | 1560 | 2054.11 | 2059.12 | 5.45 | 0.037095 |
| S2D2 | 15802 | 1218 | 1649.72 | 1617.56 | 5.20 | 0.036584 |
| S2D3 | 11995 | 1144 | 1615.76 | 1620.72 | 5.43 | 0.041665 |
| S2D4 | 25781 | 1774 | 2519.88 | 2072.01 | 5.59 | 0.033868 |
| S2D5 | 23073 | 1464 | 2014.50 | 3004.32 | 5.47 | 0.039826 |
| S3A1 | 17476 | 1731 | 3759.20 | 3004.32 | 5.76 | 0.012303 |
| S3A2 | 21493 | 2374 | 5523.92 | 4279.10 | 6.06 | 0.009494 |
| S3A3 | 17241 | 1804 | 4045.17 | 3261.04 | 5.87 | 0.010407 |
| S3A4 | 16145 | 1787 | 3879.21 | 3078.04 | 5.91 | 0.009781 |
| S3A5 | 10440 | 1293 | 3054.41 | 2451.68 | 5.58 | 0.014801 |
| S3B1 | 15249 | 1510 | 2934.07 | 2482.40 | 5.56 | 0.013753 |
| S3B2 | 22748 | 2030 | 4947.38 | 3865.68 | 5.65 | 0.01444 |
| S3B3 | 41322 | 2406 | 5762.57 | 4375.73 | 5.46 | 0.017133 |
| S3B4 | 28824 | 1774 | 3460.60 | 2901.22 | 5.40 | 0.016093 |
| S3B5 | 32239 | 2525 | 7616.72 | 5448.05 | 5.58 | 0.014879 |
| S3C1 | 28242 | 2644 | 6911.76 | 5259.20 | 5.83 | 0.013713 |
| S3C2 | 33153 | 2364 | 5058.48 | 4056.15 | 5.77 | 0.012811 |
| S3C3 | 31688 | 1972 | 4037.90 | 3518.12 | 5.43 | 0.019248 |
| S3C4 | 31662 | 2311 | 5322.62 | 4342.87 | 5.67 | 0.014242 |
| S3C5 | 11102 | 1369 | 3049.54 | 2486.96 | 5.44 | 0.020041 |
| S3D1 | 17120 | 1724 | 4967.74 | 3461.80 | 5.55 | 0.013044 |
| S3D2 | 15986 | 1300 | 2649.47 | 2116.53 | 5.33 | 0.015447 |
| S3D3 | 42607 | 2386 | 6674.56 | 5299.55 | 5.54 | 0.013875 |
| S3D4 | 34125 | 2150 | 5555.73 | 4220.29 | 5.49 | 0.015631 |
| S3D5 | 45430 | 2691 | 7655.58 | 5470.60 | 5.62 | 0.013229 |

^a^S1, S2, and S3 represent 52 d, 62 d, and 72 d; A, B, C, and D represent different diets, and 5 repeats per diet treatment were named 1, 2, 3, 4, and 5, respectively.
